# Supplementary material for: Treating Critically Ill Patients Experiencing SARS-CoV-2 Severe Infection with Ig-M and Ig-A Enriched Ig-G Infusion
Source: Antibiotics (Basel). 2021 Jul 30;10(8):930. doi: 10.3390/antibiotics10080930 (PMC8388937; doi:10.3390/antibiotics10080930)
Supplement: Supplementary file 1 [file antibiotics-10-00930-s001.zip › antibiotics-1248250-supplementary-done.pdf]

antibiotics

Supplementary Materials

Treating Critically Ill Patients Experiencing SARS-CoV-2 Severe Infection with Ig-M Enriched Ig-G Infusion

Alberto Corona MD *, Giuseppe Richini, Sara Simoncini, Marta Zangrandi, Monica Biasini, Giuseppe Russo, Mauro Pasqua, Camilla Gregorini and Chiara Giordano

Accident & Emergency and Anaesthesia and Intensive Care Medicine Department, Esine and Edolo Hospitals, ASST Valcamonica, 25043 Brescia, Italy

Supplementary Material 1

In the following tables we show all the recorded and analysed anamnestic, clinical, bioumoral, instrumental and ventilation parameters, both stratified according with groups and outcome.

Table S1. Quantitative variables stratified between treated and not treated.

Variables	IgM-Enriched IgG Treated	Not Treated	*p*-Value
Age (yrs.)	59.5 (57-66)	61(55-67)	0.212
SAPS II (score)	45 (38-59)	46 (38-58)	0.222
SOFA (score)	9 (7-10)	8 (5-11)	0.174
Pre-ICU-LOS (days)	3 (1-5)	4 (1-10.5)	0.620
ICU-LOS (days)	15.5 (4.5-15.5)	9 (4-11)	0.045
Urea (mg/dL)	55 (40-109)	48 (30-74)	0.209
Total bilirubin (mg/dL)	0.9 (0.6-1.9)	1 (0.8-1.7)	0.529
Ammonium (mg/dL)	76 (57-109)	72 (53-100)	0.335
Total diuresis (L/die)	1.7 (1.2-2.2)	1.7 (1.1-2.6)	0.202
PLT	$110*10^3$(75-275)	$105*10^3$ (86-233)	0.358
LDH (mg/dL)	525 (320-705)	462 (272-563)	0.478
CPK (mg/dL)	2239 (1140-2537)	3467 (2228-4185)	0.022
D-Dimer (µg/dL)	6485(1400-11.000)	10.7 (1.200-20000)	0.110
Worse CRP (mg/dL)	265 (165-370)	226 (87-287)	0.087
Worse PCT (ng/dL)	3.6 (0.6-7.4)	4.16 (0.5-8.9)	0.075
Worse WBC (mg/dL)	$20.2*10^3$(6.4-32.3)	$19.3*10^3$ (5.9-27.2)	0.334
Ferritinaemia (mg/dL)	5.9 (1.2-7.8)	7.0 (1.3-8.1)	0.447
Albumin (g/L)	2.3 (2.1-3.8)	2.2 (2.1-3.6)	0.652
Worst Temperature (°C)	39.5 (37.8-40.1)	39.3 (37.7-39.8)	0.233
IgG-pre (mg/dL) [range 7.5-15.6]	10 (7-14.5)	10.6 (6.8-14.6)	0.233
IgG-post (mg/dL) [range 7.5-15.6]	11.4 (7.6-15.4)	9.8 (7.8-14)	0.065
IgA-pre (mg/dL) [range 0.82-4.53]	0.8 (0.7-3.1)	0.9 (0.8-3)	0.125
IgA-post (mg/dL) [range 0.82-4.53]	1.8 (1-3.8)	1 (0.75-1.9)	0.086
IgM-pre (mg/dL) [range 0.46-3.04]	0.5 (0.4-1.5)	0.6 (0.4-1.6)	0.276
IgM-post (mg/dL) [range 0.46-3.04]	1.2 (0.6-2.6)	0.7 (0.5-1.9)	0.071
TC involvement (%)	75 (45-88.75)	65 (30-72.5)	0.115
Recruiting Maneouvres (#)	1 (1-2)	1 (1-2)	1
Muscle block duration (days)	3.5 (2-6)	3 (2.5-5.5)	0.122
Min-PEEP (cmH_2O)	10 (5-14)	10.5 (8-12.5)	0.224
Max-PEEP (cmH_2O)	16 (15-18)	15 (13.5-18)	0.405

Worse PaO_2/FiO_2 ratio (#)	116 (86-176)	120 (74-230)	0.725
pre intubation			
pH (#)	7.48 (7.37-7.48)	7.47 (7.42-7.49)	0.9
pCO2 (mmHg)	34 (30-38)	36 (28-40)	0.1
PaO2/FiO2 (#)	84 (66-166.5)	110 (63-157)	0.09
Lactates (mMol/L)	2.3 (1.5-3)	1.6 (1.2-2.5)	0.068
post intubation			
pH (#)	7.37 (7.31-7.42)	7.38 (7.28-7.46)	0.212
pCO2 (mmHg)	42 (37-47)	43 (32-52)	0.234
PaO2/FiO2 (#)	155 (105-191)	165 (116-205)	0.181
Lactates (mMol/L)	1.4 (1.2-1.98)	1.4 (1.1 – 2.7)	0.460
PEEP (cmH_2O)	15 (13.75-16.5)	12.5 (11.75-15)	0.613
1st pronation			
pH (#)	7.32 (7.25-7.42)	7.26 (7.20-7.34)	0.246
pCO2 (mmHg)	46 (45-61)	50 (43-73)	0.659
PaO2/FiO2 (#)	127 (97-162)	210 (126-288)	0.328
Lactates (mMol/L)	1.9 (1.6-2)	1.6 (1.1-2.6)	0.659
PEEP (cmH_2O)	15 (13.75-16.5)	12.5 (11.75-15)	0.613
1st re-supination			
pH (#)	7.32 (7.20-7.42)	7.33 (7.26-7.4)	1
pCO2 (mmHg)	48 (39-61)	51 (44-61)	0.681
PaO2/FiO2 (#)	142 (100-202)	181 (91-218)	0.837
Lactates (mMol/L)	2.2 (1.9-2.7)	1.9 (1.1-2.2)	0.174
PEEP (cmH_2O)	12 (10-16)	12 (10-14)	0.545
2nd pronation			
pH (#)	7.38 (7.33-7.43)	7.35 (7.33-7.37)	0.476
pCO2 (mmHg)	48 (36-57)	52 (43-56)	0.914
PaO2/FiO2 (#)	174 (125-233)	89 (72-223)	0.044
Lactates (mMol/L)	1.7 (1.4-2.6)	1.3 (1.2-2.6)	0.476
PEEP (cmH_2O)	12 (10-14)	12 (10-14)	1
2nd re-supination			
pH (#)	7.43 (7.39-7.45)	7.37 (7.31-7.53)	0.475
pCO2 (mmHg)	45 (42-50)	50 (34-64)	0.914
PaO2/FiO2 (#)	132 (70-107)	195 (157-202)	0.048
Lactates (mMol/L)	2 (1.5-2.4)	1.8 (1-2.2)	0.610
PEEP (cmH_2O)	10 (10-12)	10 (8-12)	0.884
Worse GCS (#)	15 (15-15)	15 (15-15)	1
MV-length (days)	12 (9-15)	7 (5-9)	0.045
Tracheostomy timing (days)	12 (7-18)	12 (6.75-15)	0.225
Expected Mortality (days)	34 (30-43)	35 (22-45)	0.137

Legend: (#): absolute number.

Table S2. Categorical variables stratified between treated and not treated.

Variables	IgM-enriched IgG Treated	Not Treated	OR (95% CI)	*p*-Value
IDDM/NIDDM	2 (8.3%)	2 (8.7%)	1 (0.2-7.9)	1
Smoke	2 (8.3%)	0	n.a.	0.488
COPD & other lung pathologies	2 (8.3%)	4 (17.4%)	0.4 (0.1-2.6)	0.416
Chronic Renal failure	1 (4.2%)	0	n.a.	1
Chronic Liver failure	0	0	n.a	n.a.

Steroid therapy & immunosuppression	0	2 (8.7%)	n.a.	0.234
Hypertension	12 (50%)	7 (33.3%)		
CAD	4(16.7%)	1 (4.3%)	4.7 (0.5-6.2)	0.343
Other Cardiopathies	2 (8.3%)	2 (8.7%)	0.9 (0.1-7.4)	1
Cerebral Stroke	1 (4.2%)	0	n.a.	1
Neoplasia	1 (4.2%)	0	n.a.	1
Vasoactive drugs	17 (70.8%)	13 (56.5%)	2.6 (0.5-12.4)	0.273
Antiviral Therapy (*)				
Hydroxychloroquine	7 (29.2%)	10 (43.5%)	0.6 (0.2-1.9)	0.530
Lopinavir/ritonavir	6 (25%)	5 (21.7%)	1.3 (0.3-5.1)	1
Steroids	3 (12.5%)	10 (43.5%)	0.2 (0.1-0.9)	0.043
Azitromicin	7 (29.2%)	6 (26.1%)	1.3 (0.3-4.6)	1
ASA	2 (8.3%)	2 (8.7%)	1 (0.2-7.9)	1
Heparin sodium 4000 IU/daily	4 (16.7%)	5 (21.7%)	0.7 (0.2-3.1)	0.719
Heparin sodium 4000 IU b.i.d.	1 (4.2%)	1 (4.3%)	1 (0.05-17.1)	1
Heparin calcium 25.000 IU/daily	1 (4.2%)	1 (4.3%)	1 (0.05-17.1)	1
O.A.T.	1 (4.2%)	0	n.a.	1
Pulmonary Embolism	3 (12.5%)	6 (26.1%)	0.4 (0.2-1.8)	0.277
Acute Kidney failure	10 (41.7%)	3 (13%)	5.1 (1.2-23)	0.043
CRRT	4 (16.7%)	1 (4.3%)	4.9 (0.5-48.4)	0.185
Acute liver failure	10 (41.7%)	5 (21.7)	2.7 (0.8-10.2)	0.197
Acute cerebral stroke	2 (8.3%)	2 (8.7%)	0.9 (0.1-7.5)	1
Infection rate	5 (20.8%)	9 (39.1%)	0.6 (0.1-1.3)	0.116
Septic shock	5 (20.8%)	7 (21.7%)	0.7 (0.2-5)	0.329

Legend: (*) pre-admission to ICU; in case of presence of slash /: at least 1 variable in each two way table upon which measures of association are computed is a constant; OR: the Mantel-Haenszel statistic is applied as well as the conditional assumption of independence. OR are computed as IgM-enriched IgG treated vs. not treated. Data of categorical variables are given in percentage.

Table 3. Quantitative variables stratified between treated and not treated and according with the outcome.

	**IgM Enriched IgG**			**Not Treated**		
**Variables**	**Survived**	**Dead**	***p*-Value**	**Survived**	**Dead**	***p*-Value**
Age (yrs.)	58.5 (55-61.5)	63.5 (58-75)	0.051	56 (48-66)	66 (63-71)	0.043
BMI (#)	27 (24-30)	31 (58-75)	0.095	24 (24-29)	27 (24-29)	0.387
SAPS II (score)	42 (38-51)	43.5 (39-48)	0.605	46 (37-55)	47 (46-62)	0.331
SOFA (score)	9 (7-10)	9 (7.5-10)	0.941	8 (7-10)	9 (8-12)	0.080
Pre-ICU-LOS (days)	3 (1-5)	1 (1-6.5)	0.917	4 (2-7)	7.5 (1-10)	0.539
ICU-LOS (days)	12 (5-18.5)	10 (4-15)	0.563	7 (6-15)	5.5 (3-11)	0.851
Urea (mg/dL)	40 (35-72)	62 (47-115)	0.095	50 (29-76)	60 (44-80)	0.360
Total bilirubin (mg/dL)	0.87 (0.65-2.37)	1 (0.6-1.6)	0.824	1.6 (0.8-1.35)	1.7 (0.7-1.8)	0.863
Ammonium (mg/dL)	76 (57-109)	78 (65-117)	0.624	52 (46-59)	94 (72-107)	0.036
PLT (#)	$236*10^3$ (198-275)	$175*10^3$ (145-240)	0.176	$183*10^3$ (140-281)	$140*10^3$ (91-192)	0.123
LDH (mg/dL)	443 (321-606)	569 (519-766)	0.112	319 (229-479)	503 (436-566)	0.025
CPK (mg/dL)	69 (24-484)	245 (92-728)	0.370	73 (30-273)	93 (23-185)	0.863
D-Dimer (μg/dL)	5140 (770-13570)	1200 (965-2005)	0.360	1770 (620-11080)	14950 (2780-20000)	0.041
CRP (mg/dL)	273 (150-305)	297 (180-317)	0.552	131 (63-170)	115 (93-220)	0.654
PCT (ng/dL)	0.59 (0.13-1)	1.2 (0.15-1.9)	0.270	0.33 (0.13-1)	0.53 (0.15-4.3)	0.055

WBC (#)	8750 (6000-11300)	10300 (6730-17900)	0.656	7390 (4900-11080)	12700 (7300-18540)	0.314
Ferritinaemia (mg/dL)	1865 (785-3898)	2283 (1351-4485)	0.297	1661 (1206-2107)	4036 (1230-10933)	0.174
Albumin (g/L)	3.3 (2.9-3.8)	3.3 (3.1-3.6)	0.876	3.5 (2.9-4.1)	3.3 (3.1-3.5)	0.310
Worst Temperature (°C)	37.5 (37-38.5)	38.2 (36.9-38.8)	0.780	37 (36.8-37.9)	37 (36.8-38.3)	0.829
IgG-pre (mg/dL) [range 7.5-15.6]	10.5 (7-15)	10 (6.9-14.6)	0.443	/	/	/
IgG-post (mg/dL) [range 7.5-15.6]	11.4 (7.8-14.4)	9.8 (7.8-14)	0.554	/	/	/
IgA-pre (mg/dL) [range 0.82-4.53]	1.1 (0.7-3.1)	0.8 (0.8-2.1)	0.294	/	/	/
IgA-post (mg/dL) [range 0.82-4.53]	1.8 (1-3.8)	0.9 (0.75-1.9)	0.187	/	/	/
IgM-pre (mg/dL) [range 0.46-3.04]	1.1 (0.6-2.1)	0.9 (0.5-1.7)	0.254	/	/	/
IgM-post (mg/dL) [range 0.46-3.04]	1.2 (0.7-2)	1 (0.55-1.9)	0.234	/	/	/
TC involvement (%)	75 (45-88.75)	65 (30-72.5)	0.115	50 (25-70)	55 (30-78)	0.418
Recruiting Maneouvres (#)	2 (2-4)	4 (2-4)	0.980	2 (2-4)	4 (2-4)	0.980
Prono-supinations (#)	2 (2-3)	2 (2-3)	0.988	2 (2-3)	2 (2-3)	0.988
Muscle block duration (days)	3 (3-5)	4 (1-6)	0.930	2 (2-3)	2 (2-3)	0.988
Min-PEEP (cmH_2O)	14 (13-15)	14 (13-15)	0.752	11 (8-14)	12 (9-12.5)	0.489
Max-PEEP (cmH_2O)	16 (15-18)	18 (15-20)	0.206	15 (12-18)	15 (13-18)	0.730
Worse PaO_2/FiO_2 ratio (#)	137 (82-106)	92 (92-111)	0.010	119 (85-180)	85 (57-139)	0.040
Worse GCS (#)	15 (15-15)	15 (15-15)	1	15 (15-15)	15 (15-15)	1
pre intubation<br>pH (#)<br>pCO2 (mmHg)<br>PaO_2/FiO_2 ratio (#)<br>Lactates (mMol/L)	 7.44 (7.43-7.48)<br>54 (42-68)<br>129 (99-197)<br>1.6 (1.2-2.4)	 7.38 (7.27-7.49)<br>38 (33-41)<br>66 (50-76.5)<br>3 (2.2-6.7)	 0.497<br>0.065<br>0.001<br>0.013	7.47 (7.43-7.50)<br>37 (27-41)<br>151 (105-190)<br>1.2 (1-1.8)	 7.38 (7.39-7.49)<br>32 (28-39)<br>91 (53-117)<br>2.2 (1.6-3)	 0.654<br>0.756<br>0.029<br>0.016
post intubation<br>pH (#)<br>pCO2 (mmHg)<br>PaO_2/FiO_2 ratio (#)<br>Lactate (mMol/L)<br>PEEP cmH_2O)	 7.39 (7.35-7.43)<br>38 (37-46)<br>182 (134-191)<br>1.3 (1-1.6)<br>15 (12-16)	 7.33 (7.26-7.39)<br>45 (42-49)<br>110 (70-190)<br>1.95 (1.5-3.7)<br>15.5 (14-18)	 0.021<br>0.083<br>0.146<br>0.019<br>0.860	7.44 (7.36-7.52)<br>37 (26-40)<br>230 (110-240)<br>1.2 (1-2.1)<br>15 (12-15)	 7.32 (7.25-7.43)<br>44 (41-54)<br>119 (82-170)<br>1.7 (1.3-3)<br>12 (10-15)	 0.012<br>0.020<br>0.016<br>0.230<br>0.515
1st pronation<br>pH (#)<br>pCO2 (mmHg)<br>PaO_2/FiO_2 ratio (#)<br>Lactate (mMol/L)<br>PEEP (cmH_2O)	 7.39 (7.25-7.47)<br>45 (40-60)<br>165 (115-235)<br>1.7 (1.5-2.3)<br>15 (12-16)	 7.27 (7.18-7.36)<br>52 (44-62)<br>118 (82.5-155)<br>1.8 (1.3-2.7)<br>15.5 (14-18)	 0.082<br>0.792<br>0.177<br>0.792<br>0.860	7.27 (7.18-7.37)<br>68 (50-85)<br>170 (126-220)<br>1.8 (1-2.6)<br>15 (12-18)	 7.27 (7.21-7.33)<br>49 (39-62)<br>126 (80-220)<br>1.7 (1.2-2.6)<br>15 (14-16)	 1<br>0.301<br>0.857<br>0.857<br>0.755
1st re-supination<br>pH (#)<br>pCO2 (mmHg)<br>PaO_2/FiO_2 ratio (#)<br>Lactates (mMol/L)<br>PEEP (cmH_2O)	 7.40 (7.28-7.50)<br>40 (28-60)<br>192 (140-225)<br>1.9 (1.6-2.2)<br>15 (12-16)	 7.27 (7.15-7.38)<br>51 (46-63)<br>102 (80-165)<br>2.7 (2.3-3)<br>15.5 (14-18)	 0.111<br>0.286<br>0.111<br>0.063<br>0.860	 7.33 (7.26-7.40)<br>60(54-66)<br>257 (137-370)<br>1.1 (0.75-1.45)	 7.33 (7.29-7.38)<br>50 (43-56)<br>181 (67-202)<br>2.2 (1.4-3.1)<br>15 (14-16)	 0.857<br>0.190<br>0.571<br>0.190<br>0.755

				15 (12-18)		
2nd pronation						
pH (#)	7.40 (7.34-7.47)	7.36 (7.30-7.42)	0.700	7.33 (7.33-7.33)	7.36 (7.34-7.38)	0.500
pCO2 (mmHg)	48 (37-53)	57 (34-58)	0.400	51 (50-55)	52 (39-57)	1
PaO_2/FiO_2 ratio (#)	138 (86-245)	200 (150-225)	0.700	139 (110-150)	197 (115-330)	0.500
Lactates (mMol/L)	1.8 (1.2-2.9)	1.9 (1.4-2.4)	1	1.2 (1.1-2)	1.9 (1.3-3.0)	0.500
PEEP (cmH_2O)	15 (14-16)	15.5 (14-18)	0.860	15 (12-18)	15 (14-16)	0.755
2nd re-supination						
pH (#)	7.44 (7.40-7.45)	7.43 (7.36-7.45)	0.700	7.30 (7.30-7.33)	7.44 (7.32-7.43)	0.550
pCO2 (mmHg)	44 (37-55)	45.7 (45-47.8)	0.700	66 (57-70)	45 (30-55)	0.478
PaO_2/FiO_2 ratio (#)	137 (82-106)	100 (96-150)	0.400	200 (180-220)	102 (95-138)	0.500
Lactates (mMol/L)	1.9 (1.1-3)	2.2. (1.9-2-2)	0.700	1 (0.8-2)	1.9 (1.7-2.3)	0.500
PEEP (cmH_2O)	14 (12-16)	14 (12-16)	1	14 (12-15)	14 (12-15)	1
MV-length (days)	11 (4-19)	9 (4-13)	0.186	10 (5-18.5)	8 (7-11)	0.043
Tracheostomy timing (days)	16.5 (9-27)	11 (5-13)	0.229	7 (6-11)	6 (5-7)	0.778
Expected Mortality (days)	30 (21-48)	30 (23-42)	0.605	32 (17-41)	34 (30-39)	0.310

Table S4. Categorical variables stratified between treated and not treated and according with the outcome.

	**IgM Enriched IgG Treated**				**Not Treated**			
**Variables**	**Survived**	**Dead**	**OR (95% CI)**	***p*-Value**	**Survived**	**Dead**	**OR (95% CI)**	***p*-Value**
IDDM/NIDDM	1 (7.1%)	1 (10%)	0.7 (0.1-13.4)	1	1 (10%)	1 (7.7%)	1.1 (0.1-20.5)	1
Smoke	2 (14.3%)	1 (10%)	2 (0.6-2.2)	0.486	0	2 (15.4%)	0.4 (0.1-4.2)	0.604
COPD & Other lung pathologies	2 (14.3%)	0	n.a.	0.493	1 (10%)	3 (23.1%)		
Chronic Renal failure	0	1 (10%)	n.a.	0.429	0	0	n.a.	n.a.
Chronic Liver failure	0	0	n.a.	n.a.	0	0	n.a.	n.a.
Steroid therapy & Immunosuppression	0	0	n.a.	n.a.	0	2 (15.4%)	n.a.	0.486
Hypertension	7 (50%)	5 (50%)	1.2 (0.2-6.4)	1	2 (20%)	5 (38.5%)	0.3 (0.1-2.1)	0.361
CAD	2 (14.3%)	2 (20%)	0.7 (0.1-6.2)	1	0	1 (7.7%)	n.a.	1
Other cardiopathies	1 (7.1%)	1 (10%)	0.7 (0.1-12.6)	1	1 (10%)	1 (7.7%)	1.3 (0.1-24.3)	1
Cerebral Stroke	0	1 (10%)	n.a.	0.429	0	2 (15.4%)	n.a.	1
Neoplasia	0	1 (10%)	n.a.	0.429	0	2 (15.4%)	n.a.	1
Vasoactive drugs	8 (57.1%)	9 (90%)	0.6 (0.1-7.1)	0.218	4 (40%)	9 (69.2%)	0.1 (0.05-1.05)	0.057
Antiviral Therapy								
Hydroxychloroquine	4 (28.6%)	3 (30%)	1 (0.1-6.2)	1	5 (50%)	5 (38.5%)	1.2 (0.2-6.7)	1
Lopinavir/ritonavir	3 (21.4%)	3 (30%)	0.7 (0.1-4.5)	1	3 (30%)	2 (15.4%)	1.9 (0.3-14.9)	0.635
Steroids	1 (7.1%)	2 (20%)	0.4 (0.1-4.7)	0.566	5 (50%)	5 (38.5%)	1.2 (0.2-6.7)	1
Azitromicin	3 (21.4%)	4 (40%)	0.4 (0.1-2.7)	0.397	2 (20%)	4 (30.8%)	0.4 (0.1-3.2)	0.635
ASA	1 (7.1%)	1 (10%)	0.7 (0.1-13.5)	1	0	2 (15.4%)	n.a.	0.476
Heparin sodium 4000 IU/daily	1 (7.1%)	3 (30%)	0.2 (0.2-2.2)	0.272	3 (30%)	2 (15.4%)	1.7 (0.3-13.4)	1
Heparin sodium 4000 IU b.i.d.	1 (7.1%)	0	n.a.	1	0	2 (15.4%)	n.a.	1
Heparin calcium 25.000 IU/daily	1 (7.1%)	0	n.a.	1	1 (10%)	2 (15.4%)	0.4 (0.1-3.2)	0.476
**O.A.T.**	1 (7.1%)	0	n.a.	1	0	2 (15.4%)	n.a.	1
Pulmonary Embolism	2 (14.3%)	1 (10%)	1.6 (0.2-21)	0.612	2 (20%)	4 (30.8%)	0.5 (0.1-3.7)	0.642
Acute Kidney failure	4 (28.6%)	6 (60%)	0.3 (0.1-1.6)	0.198	1 (10%)	2 (15.4%)	0.6 (0.1-7.4)	0.479
CRRT	3 (21.4%)	1 (10%)	2.7 (0.3-31)	0.603	0	1 (7.7%)	n.a.	1
Acute liver failure	4 (28.6%)	6 (60%)	0.3 (0.1-1.6)	0.198	1 (10%)	4 (30.8%)	0.2 (0.1-2.4)	0.319
Cerebral strokes	1 (7.1%)	1 (10%)	0.7 (0.1-13.5)	1	1 (10%)	2 (15.4%)	0.6 (0.1-7.4)	0.479
Infection rate	1 (7.1%)	3 (23.1%)	0.3 (0.1-3.8)	0.596	5 (50%)	4 (30.8%)	1.1 (0.1-5.1)	0.637
Septic shock	1 (7.1%)	4 (40%)	0.2 (0.1-2.4)	0.327	4 (40%)	3 (23.1%)	1.1 (0.2-6.2)	0.663

Legend: in case of presence of slash /: at least 1 variable in each two way table upon which measures of association are computed is a constant; OR: the Mantel-Haenszel statistic is applied as well as the conditional assumption of independence. Data of categorical variables are given in percentage.

Supplementary Material 2

Methods S1: Variable life adjustment display

Variable life adjustment display (VLAD), is a type of indicator used to measure healthcare quality and patient outcomes (1). VLAD allows to ascertain the difference between expected mortality [indicated by SMR (standardised mortality rate) derived from SAPS II and recorded mortality]

1st 24 hrs. SAPS II is an all over the world well known and used score (2). Intensivists apply it to assess the probability of death of the patients upon the ICU admission. The score values range from 0 up to 150 (0 up to 100% of death probability). The increasing of death probability is exponential, according with the increasing of SAPS II. On the other hand, death probability upon ICU admission derived from SAPS II does not allow intensivist to take the decision of not to treat patients.

Once calculated the SAPS II intensivists may derived SMR (standardised mortality rate) that ranges from 0 to 1 and indicates the probability of death of that kind of patients meanwhile he/she is admitted to ICU.

SMR is calculated from SAPS II from the following exponential equation:

Hospital mortality may be calculated using the following equations (2):

$$\text{logit} = -7.7631 + 0.0737 * (\text{SAPS II}) + 0.9971 * \ln(\text{Score}+1)$$

$$\text{Mortality (SMR)} = \frac{e\,logit}{1 + e\,logit}$$

Following on this we computed VLAD (1) to assess differences between expected and observed deaths.

VLAD

Example A): SMR is < 0.5, i.e., = 0.15, i.e., 15% of death likelihood, considering that such a mortality is very low:

A_1) if the patient survives, we gain 0.15 points of life corresponding to the death likelihood, therefore VLAD = +0.15

A_2) if the patient dies, we lose points of life corresponding to the survive probability, therefore VLAD = - 0.85

Example B): SMR is > 0.5, i.e., = 0.75, i.e. 75% of death likelihood, considering that such a mortality is very high:

B_1) if the patient survives, we gain 0.75 points of life corresponding to the death likelihood, therefore VLAD = + 0.75

B_2) if the patient dies, we lose points of life corresponding to the survive probability, therefore VLAD = - 0.15

Considering a number of patients admitted to an ICU we can sum up each VLAD of each patients; so far, we obtain a definitive a cumulative VLAD. If it is positive: observed mortality is higher than expected one, otherwise not. Moreover, in case of positive cumulative VLAD, cumulative lives saved in ICU are higher than those expected and predicted by SAPS II, otherwise not. In the former situation we can assess that ICU performance was better than in the latter one (negative cumulative VLAD).

Supplementary Material 3

Methods 3: The Posology Algorithm

IgM/IgA enriched IgG are given at the dosage of 250 mg/ kg to be infused over 72 h.

The choice of dosage was made on the basis of a severity score summarised in the below table S5, calculated on the basis of the severity of the clinical picture, the basal serum values of Immunoglobulins and CD 4+, 8+ Lymphocytes, and according to the timing of the start of treatment with respect to onset of symptoms.

Table S5. Severity SCORE to implement the proper posology.

IgA-G-M	SCORE
• BETWEEN NORMALITY RANGE	0
• UNDER NORMALITY RANGE	2
LYMPHOCYTES CD 4+ - 8+	SCORE
• BETWEEN NORMALITY RANGE	0
• UNDER NORMALITY RANGE	2
DELTA-TIME FROM CLINICAL ONSET TO TREATMENT STARTING	SCORE
• < 12 ore	0
• 12 – 24 ore	1
• ≥ 24 ore	2
SEVERITY OF ORGAN FAILURE	SCORE
•4 ≤ SOFA < 6	0
• 6 ≤ SOFA < 8	1
• SOFA ≥ 8	2

For SCORE ≤ 2: IgM/IgA-enriched IgG at a dosage of 250 mg/kg over 72 h; For SCORE > 2 IgM/IgA-enriched IgG at a dosage of 500 mg / kg in 72 h.

In case of pre-existing renal insufficiency, we will use the baseline creatinine clearance value to proceed as follows (Table S5):

Table S6. Pentaglobin dosage in case of renal failure.

**Cr-Cl (mL/min)**	**IgM/IgA—Enriched IgG Posology (mg/kg)**
Cr-Cl ≥ 50	500
50 < Cr-Cl < 30	
SCORE < 2	250
SCORE ≥ 2	To be individualised (*)
Cr-Cl ≤ 30	250 undependentely by SCORE

Legend: (*): according with severity of clinical conditions.

References

1. S1 Foltran, F.; Baldi, I.; Bertolini, G.; Merletti, F.; Gregori, D. Monitoring the performance of intensive care units using the variable life-adjusted display: a simulation study to explore its applicability and efficiency. *J. Evaluation Clin. Pr.* **2009**, *15*, 506–513, doi:10.1111/j.1365-2753.2008.01052.x.
2. S2 Le Gall, J.R. A new Simplified Acute Physiology Score (SAPS II) based on a European/North American multicenter study. *JAMA* **1993**, *270*, 2957–2963, doi:10.1001/jama.270.24.2957.
